# Scaling laws governing droplet spreading and merging dynamics on solid surfaces: a molecular simulation study

**DOI:** 10.1140/epje/s10189-026-00625-4

**Published:** 2026-08-26

**Authors:** Ertiza Hossain Shopnil, Jahid Emon, Md Nadeem Azad, A. K. M. Monjur Morshed

**Affiliations:** 1 Department of Mechanical Engineering, Virginia Tech, Blacksburg, VA 24061 USA; 2 Department of Mechanical Science and Engineering, University of Illinois Urbana-Champaign, Urbana, IL 61801 USA; 3 Department of Aerospace and Mechanical Engineering, University of Notre Dame, Notre Dame, IN 46556 USA; 4 Department of Mechanical Engineering, Bangladesh University of Engineering and Technology (BUET), Dhaka, Bangladesh

## Abstract

This study employs molecular dynamics simulations to investigate droplet dynamics when a stationary droplet on a solid surface is struck by another droplet of similar size from above. The focus is on the jumping behavior of the merged droplet and the associated energy conversion. The process is primarily controlled by the amount of energy converted into kinetic energy after dissipation. At high impact velocities, the energy conversion efficiency becomes constant, with only about 1% lost due to surface adhesion—an effect that diminishes with increasing velocity. Factors such as impact velocity, droplet size, surface texture, and wettability significantly influence the jumping velocity. Scaling laws are developed for the maximum spreading time, spreading factor, and restitution coefficient based on the Weber (We) and Reynolds (Re) numbers, which differ from those for single droplet impacts. On superhydrophobic surfaces, the spreading time is approximated as ${t}_{sp}\approx 3r/{V}_{i}$, and its dimensionless form scales linearly with $W{e}^{0.31}$. The general scaling law for the spreading factor is ${\upbeta}_{max}\sim W{e}^{0.5\upalpha }R{e}^{\upalpha }$, where α=0.1 for velocity-dependent and α=0.24 for velocity-independent spreading regimes.

**Graphical abstract:**

Energy Conversion and Jumping Dynamics of Colliding Nanodroplets on Solid Surfaces.

**Figure:**
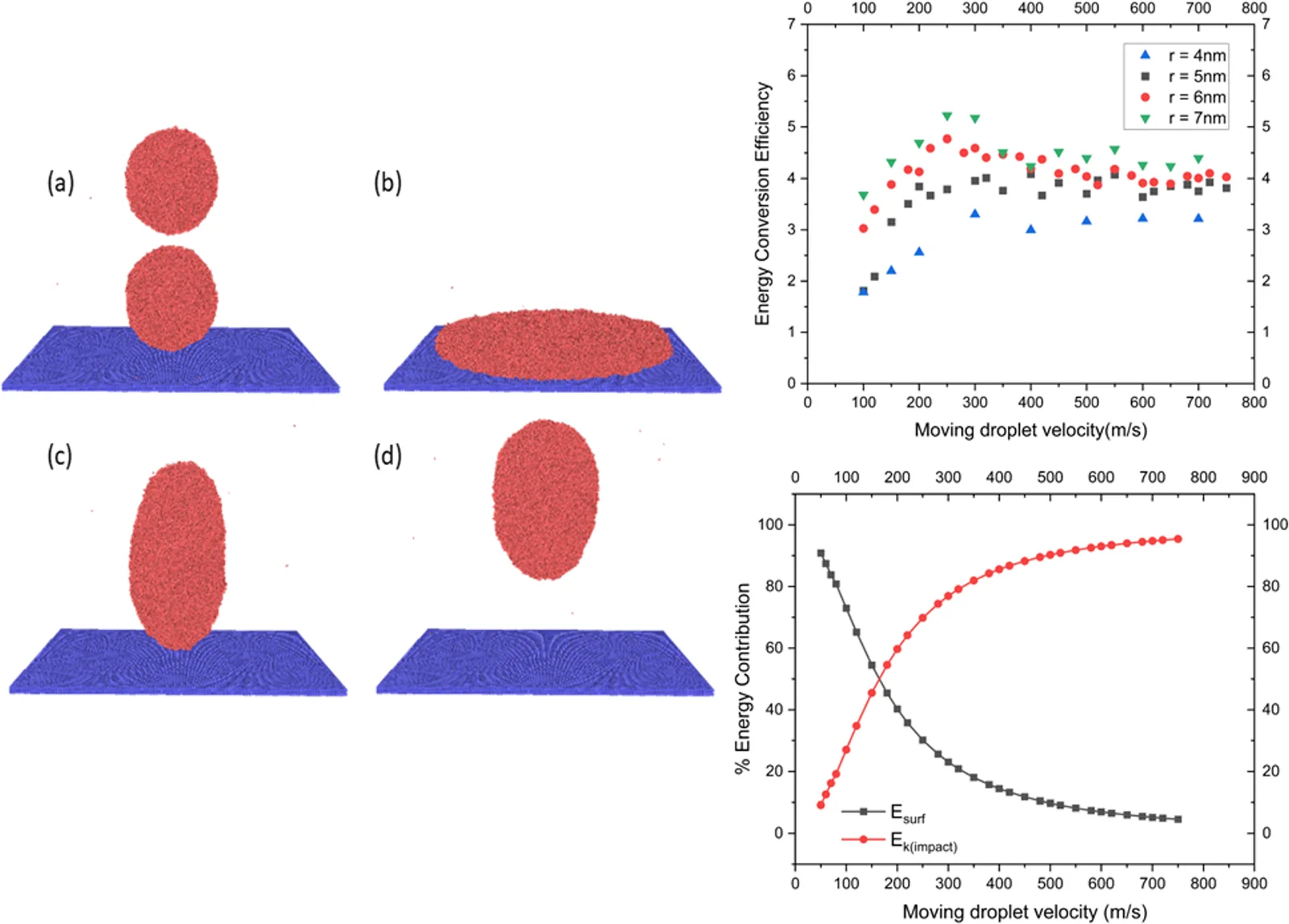

## Introduction

Droplet collision, reshaping, and bouncing are complicated process that occurs very often in both natural [1, 2] and industrial processes [3–6]. When a droplet strikes a surface, it undergoes a sequence of events: impingement, spreading, receding, and rebounding on that surface in some instances [7–9]. However, when a single droplet strikes another preexisting droplet on the surface, it undergoes an additional step known as coalescence upon impact [10]. This phenomenon has significant applications in nano-inkjet printing [11–13], surface coating [14], surface cleaning [15–19], and spray cooling [20]. Anti-icing applications in high altitudes can require insight into this phenomenon [21–23]. Additionally, tapping the renewable blue energy, such as using piezoelectricity to harvest raindrop energy, demands an understanding of the mechanism and energy conversion of droplet impact dynamics [24]. Recently, Wu et al*.* [25] have investigated the conversion of kinetic energy to electric energy when a droplet impacts a charged surface. This study presents a novel approach to energy harvesting, using droplet impact. So, the droplet impact dynamics have been the subject of recent research interest, including both experimental [26–31] and computational investigations [32–38].

Dimensionless numbers describing the droplet impact phenomena are the Weber number, $We=\frac{\uprho {D}_{0}{V}_{0}^{2}}{\upgamma }$, and Reynolds number,$Re=\frac{\uprho {D}_{0}{V}_{0}}{\upmu }$, where density $\left(\uprho \right)$, initial droplet diameter $\left({D}_{0}\right)$, impact velocity $\left({V}_{0}\right)$, surface tension $\left(\upgamma \right)$, and dynamic viscosity $\left(\upmu \right)$ are the properties that determine these dimensionless numbers. The phenomenon of a droplet impacting a substrate is generally explained with parameters such as the maximum spreading time, the maximum spreading factor, and the restitution coefficient. The time required to attain the maximum spread diameter ${D}_{max}$ while spreading upon impact is known as the maximum spreading time, where the maximum spreading factor is ${\upbeta}_{max}=\frac{{D}_{max}}{{D}_{0}}$. The impact droplet velocity ${V}_{0}$ and the induced jumping velocity ${V}_{jump}$, which is the velocity the droplet obtains from the remaining kinetic energy after viscous dissipation and work of adhesion, are used to measure the restitution coefficient $\upepsilon =\frac{{V}_{jump}}{{V}_{0}}$. Using these parameters, both experimental [39–42] and numerical [43–47] investigations are conducted to characterize impact droplet dynamics. Koishi et al*.* [35] identified the deformation of droplets necessary to bounce back from flat and nano-structured surfaces at the nanoscale with the help of molecular dynamics (MD). As the accuracy of macroscopic models drops in the case of nanoscale, Gao et al. [36] utilized MD to develop a model for a single droplet impacting a rough surface. Following this work, Wang et al. [37] developed scaling laws governing the maximum spreading factor for single droplet impact.

While numerous studies explain the phenomenon of a single droplet impacting a substrate, there is a lack of equivalent investigations concerning the collision that occurs when the impacting droplet hits a preexisting droplet on the surface. In practical applications such as nano-inkjet printing and spray cooling, where multiple droplets are deposited sequentially onto a surface, the probability of an impacting droplet encountering a preexisting surface droplet is non-negligible and increases with surface coverage. Therefore, a systematic study to understand this phenomenon is required to simulate more practical situations of droplet dynamics and adapt to engineering applications, such as nanoscale devices, particularly for nano-injection applications [11]. Xu et al. [31] investigated the phenomenon by varying the temperature of the impacting droplet while keeping the temperature of the surface droplet constant, aiming to determine the optimal temperature for the rebounding characteristics of the merged droplet.

However, in previous studies, no comprehensive analysis has been conducted for a droplet striking another immobile droplet on a rigid surface, and the effects of droplet size, surface roughness, and wettability on this phenomenon. This study presents a quantitative analysis at the nanoscale using MD simulation, revealing insights into energy conversion, droplet dynamics, and previously unexplored implications of droplet size, surface roughness, and wettability. A comparison of this phenomenon with traditional studies of a single droplet striking a surface is developed based on different factors. Through examining the interactions, modified scaling laws have been developed for important governing parameters like maximum spreading time, spreading factor, and restitution factor. These findings from the research provide a foundation for nano-injection technologies and the design of novel devices that utilize droplet collisions over surfaces.

## Methodology

**Fig. 1:**
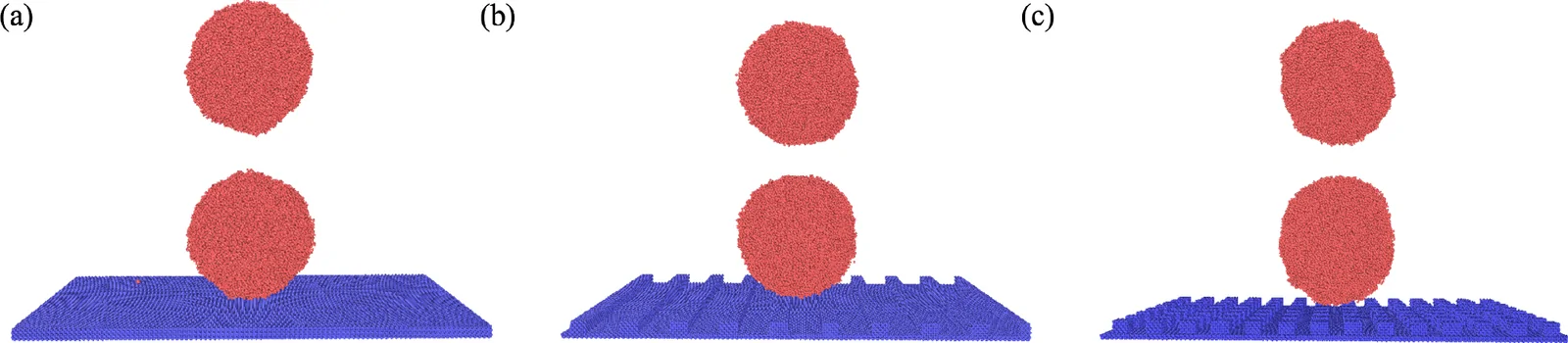
Initial configuration of the simulation domain, for the droplet on a **a** flat surface, **b** grooved surface, and **c** the surface containing nano-pillars (NP)

Molecular dynamics simulations were performed with the LAMMPS package (23 June 2022 stable release) [48], and the initial geometry was generated by Atomsk [49]. The simulation model with a flat surface initially is shown in Fig. 1a. To evaluate the surface roughness effect, two types of structures were constructed on the flat surface of Fig. 1a: grooves and nano-pillars (NP), shown in Fig. 1b and c, respectively. The initial configuration consisted of a three-dimensional box with periodic boundary conditions applied to all the boundaries. Copper (Cu) has been chosen as the substrate, modeled with a 3.61 Å lattice constant as a face-centered cubic beneath the droplets. The substrate size was optimized to accommodate the highest spreading of the merged droplet after collision. The radius of the droplets ranged from 3 nm to 7 nm. Based on the Tolman equation with δ ≈ − 0.056 nm for the mW model at 300 K [50], the finite-size correction to the bulk surface tension ranges from approximately 1.6% for r = 7 nm to 3.9% for r = 3 nm. The use of the bulk surface tension value is therefore justified for the droplet sizes considered here and is not expected to significantly alter the reported scaling laws. For the grooved surface, the height and thickness of each groove were 0.7 nm and 1.2 nm and the gaps between adjacent grooves were 3 nm. For the surface containing nano-pillars, the height and width of each nano-pillar were 0.7 nm and 1.2 nm. Three times the droplet radius was chosen as the vertical distance between the droplets’ centers to minimize simulation time and enable clear observation of the phenomenon.

The coarse-grained water model, or mW model, was used in the simulation to simulate the water nano-droplets [51]. The mW properties of water needed for calculating the Ohnesorge number (Oh), Reynolds number (Re), and Weber number (We) were taken from a recent study [52]. The Stillinger–Weber potential was used to describe interactions between mW atoms in the mW model [51]. (Table 1a) At 300 K and 1 atm, the mW density is 1000 kg/m [3], the mW viscosity is 310 *µPa·s*, and the mW surface tension is 65.4 mN/m. [52] The mW model considers oxygen and hydrogen as a single molecule to reproduce the nanoscale fluid dynamics of real water without the hydrogen atoms’ reorientation. This is reflected in the mW model’s viscosity, which is three times lower than that of actual water [51, 53]. The mW model has previously been used in studies of droplet coalescence that highlight its applicability in the MD simulation of this study [54, 55]. The Lennard–Jones potential was used to describe interactions between other molecules, and the size parameter was set to $\upsigma =3.92 {\AA }$. (Table 1b) The energy parameter was gradually adjusted from 0.05 to 0.01 kcal/mol during equilibration to increase the hydrophobicity of the surface. The value of ϵ=0.01 kcal/mol was chosen to achieve a contact angle of approximately 180 degrees and ensure superhydrophobicity between the substrate and the water droplet. ϵ=0.02 and 0.03 kcal/mol were taken for contact angles ∼165 and ∼155 degrees, respectively. The cutoff distance was set at 13.0 Å, and the time step was adjusted based on the number of atoms in the system. Time steps of 1 fs, 5 fs, and 10 fs were simulated for several cases. The time step of 5 fs was selected for droplets up to 5 nm in size, while a time step of 10 fs was chosen for droplets larger than 5 nm, as these provided greater accuracy while optimizing computational time.

During the equilibration stage, the substrate molecules were immobilized by setting their forces to zero. The entire system was relaxed using the conjugate gradient (CG) method and equilibrated with the Berendsen thermostat at 300 K, employing damping constants of 100 times the time step for the thermostat. Subsequently, the system was equilibrated under the NVT (canonical) ensemble using the Berendsen thermostat, followed by NVE (microcanonical) integration to allow the system to relax to a stable total energy. All constraints were removed after equilibration, and an impact velocity was imposed on the upper droplet by assigning the same downward velocity to every molecule of that droplet, thereby overwriting the velocities generated during equilibration. The droplet consequently begins the impact stage as a rigidly translating body with no internal relative motion; the thermal velocity distribution is re-established within the first few picoseconds of the subsequent NVE evolution, well before contact with the stationary droplet occurs. The vertical component of the mass center’s velocity was calculated by averaging the velocity per atom. This was defined as the jumping velocity induced after the impact. Similarly, the vertical component of the force was determined by averaging the force per atom. The magnitude of the force exerted by the droplet on the wall helped to determine the time when the droplet completely detached from the surface.

**Table 1:** Potential parameters used in MD simulations

(a) Stillinger–Weber potential for mW water–water interaction										
ε (kcal/mol)	σ (Å)	a	λ	γ	cosθ₀	A	B	p	q	tol
6.189	2.3925	1.8	23.15	1.2	− 0.3333	7.0496	0.6022	4	0	0

(b) Lennard–Jones (LJ/cut) potential for wall and wall–water interactions			
Interaction	ε (kcal/mol)	σ (Å)	Cutoff (Å)
Wall–wall	0.000	3.92	13.0
Wall–water (equilibration step 1)	0.050	3.92	13.0
Wall–water (equilibration step 2)	0.040	3.92	13.0
Wall–water (equilibration step 3)	0.030	3.92	13.0
Wall–water (equilibration step 4)	0.020	3.92	13.0
Wall–water (equilibration step 5)	0.010	3.92	13.0

## Results and discussion

### Model validation

**Fig. 2:**
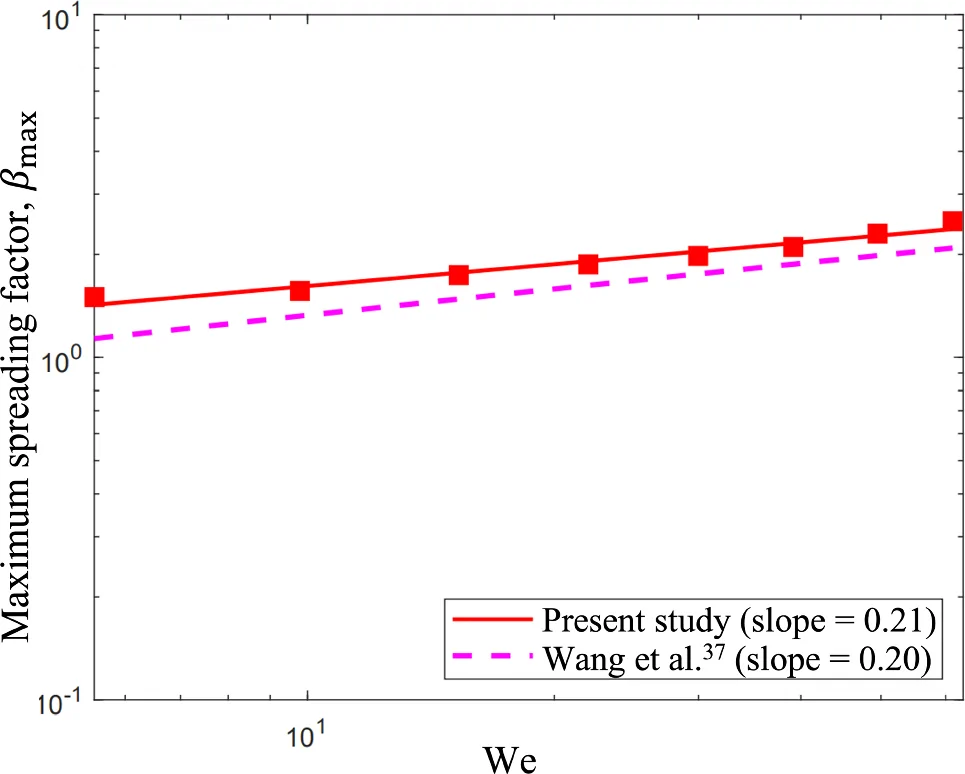
Maximum spreading factor as a function of *We* number from the present study and from Wang et al. [37] for a 14-nm-diameter droplet

To validate the present simulation model, a single droplet impact test on a superhydrophobic surface having a contact angle of nearly 180 degrees was conducted. This choice was made because the droplet dynamics for the droplet impacting another droplet on a substrate have not been explicitly studied before. Figure 2 shows the comparison of the present work to a previous study by Wang et al*.* [37] The maximum spreading factor from the present study for a 14-nm-diameter droplet matches that of Wang et al*.* [37] In both studies, the mW model was utilized to simulate water droplet behavior. Wang et al*.* [37] pointed out that for We number 1 <We< 34, the maximum spreading factor of the droplet impacting on a superhydrophobic surface follows the following scaling law:

1 $${\beta}_{\mathrm{m}\mathrm{a}\mathrm{x}}\sim W{e}^\frac{1}{5}\sim W{e}^{0.2}$$

From the present simulation for a 14-nm-diameter droplet, the power fit slopes were also found to be 0.2115.

### Jumping process and energy conversion

**Fig. 3:**
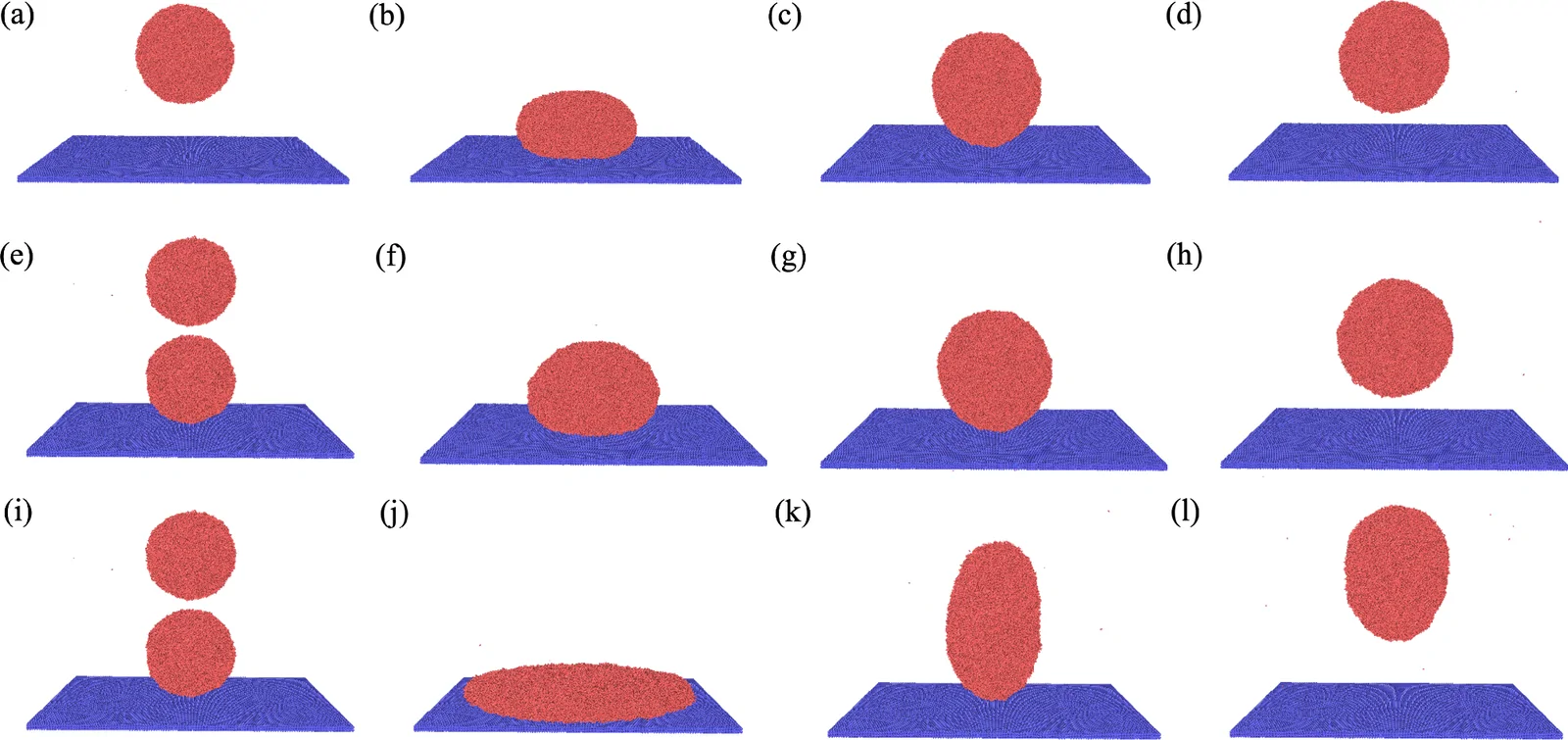
**a**–**d** Single droplet impacting on a surface. **e**, **i** Just before impacting the stationary droplet with an impact velocity of 100 m/s and 700 m/s, respectively. The total energy of this stage is the kinetic energy of the moving droplet and the surface energy of individual droplets. **f**, **j** Spreading, similar to the single droplet case. Reshaping to a **g** round stable shape, **k** unstable oval shape. The surface area of the merged droplet is less than the total surface area of the individual droplets. **h**, **l** Jumping from the surface

The collision of a stationary droplet with a moving one causes the merged droplet to spread over the surface. An upward velocity is induced as a result of the reaction force of the impact and the reshaping process of the droplet into a round shape after spreading. The process is sequentially presented in Fig. 3. Whether the induced velocity is sufficient to detach the merged droplet from the wall is determined by the droplet’s available energy after impact, which can be converted into kinetic energy. In the case of a droplet impacting another stationary droplet, the sources of energy are the kinetic energy of the moving droplet and the excess surface energy released due to the merging of the individual droplets. During the spreading of the merged droplet, kinetic energy is converted into surface energy, and at maximum spreading, the majority of the kinetic energy is converted into surface energy. At this point, the merged droplet exhibits the highest surface area and surface energy. This surface energy is released as the droplet starts reshaping into a stable round shape, characterized by minimum surface area and surface energy. So, excess surface energy is available for conversion to kinetic energy, potentially causing jumping. However, a portion of the excess surface energy is dissipated in viscous dissipation during reshaping, where a portion of the kinetic energy of the moving droplet is lost in viscous dissipation while spreading to a maximum diameter. Loss of energy in viscous dissipation is always there, whether the droplet is spreading or reshaping. Also, the present study considers the droplet radius from 3 to 7 nm, and the Oh number is quite high (0.46–0.7); hence, the loss in viscous dissipation is likewise high. Xu et al. [31] highlighted that a portion of the excess energy is also lost by internal vibration due to the oscillation of the merged droplet. The oscillation of the droplet depends on the viscous effect. Zrnic et al. [56] studied nonlinear shape oscillations of Newtonian droplets, where the oscillation frequency decreases with the Oh number increase, i.e., the viscous effect causes a large dampening of the oscillating frequency, and the damping rate rises with the Oh number. They found a cutoff of Oh 0.56 where the oscillation becomes zero. Also, while visualizing the simulation of the present work, the vibration due to the collision was hardly noticeable. So, we assume there is no significant energy loss due to droplet oscillation here. When the surface energy is fully converted to kinetic energy after the reshaping process from the spread form, the droplet does not leave the surface immediately. The maximum induced velocity is lowered by surface adhesion. So, the energy to overcome surface adhesion comes from kinetic energy. To sum up, energy is wasted in viscous dissipation and wall adhesion.

The energy balance is:

2 $${E}_{K\left(\mathrm{i}\mathrm{m}\mathrm{p}\mathrm{a}\mathrm{c}\mathrm{t}\right)}+{E}_{s1}+{E}_{s2}={E}_{K\left(\mathrm{e}\mathrm{f}\mathrm{f}\mathrm{e}\mathrm{c}\mathrm{t}\mathrm{i}\mathrm{v}\mathrm{e}\right)}+{E}_{s12}+{E}_{\mathrm{v}\mathrm{i}\mathrm{s}}+{E}_{\mathrm{a}\mathrm{d}\mathrm{h}}$$

Here, ${E}_{K\left(impact\right)}$ is the kinetic energy of the moving droplet, ${E}_{s1}+{E}_{s2}$ is the sum of the surface energies of two individual droplets before the collision, ${E}_{K\left(effective\right)}$ is the kinetic energy of the merged droplet while detaching from the surface, ${E}_{s12}$ is the surface energy of the merged droplet, ${E}_{adh}$ is the energy waste in overcoming adhesion of surface, and ${E}_{vis}$ is the energy lost in viscous dissipation.

Portion of total excess surface energy coming from the individual surface energies of the droplets after reshaping to a round shape:

3 $${E}_{\mathrm{s}\mathrm{u}\mathrm{r}\mathrm{f}}={E}_{s1}+{E}_{s2}-{E}_{s12}$$

Excess surface energy can be expressed as:

4 $${E}_{\mathrm{s}\mathrm{u}\mathrm{r}\mathrm{f}}=4\upsigma \uppi {r}^{2}\left(2-{2}^{2/3}\right)$$

where ‘σ’ is surface tension and ‘r’ is the initial radius of the droplets.

The kinetic energy of the impact droplet and the merged droplet:

5 $${E}_{K\left(\mathrm{i}\mathrm{m}\mathrm{p}\mathrm{a}\mathrm{c}\mathrm{t}\right)}=\frac{4}{6}\uppi \uprho {r}^{3}{V}_{i}^{2}$$

and

6 $${E}_{K\left(\mathrm{e}\mathrm{f}\mathrm{f}\mathrm{e}\mathrm{c}\mathrm{t}\mathrm{i}\mathrm{v}\mathrm{e}\right)}=\frac{8}{6}\uppi \uprho {r}^{3}{V}_{j}^{2}$$

where ‘r’ is the radius of the impacting droplet, ‘ρ’ is density, ${V}_{i}$ is the impact droplet velocity and ${V}_{j}$ is the final velocity of the merged droplet while detaching from the wall.

Adhesive work:

7 $${E}_{\mathrm{a}\mathrm{d}\mathrm{h}}={E}_{K\left(\mathrm{i}\mathrm{n}\mathrm{d}\mathrm{u}\mathrm{c}\mathrm{e}\mathrm{d}\right)}-{E}_{K\left(\mathrm{e}\mathrm{f}\mathrm{f}\mathrm{e}\mathrm{c}\mathrm{t}\mathrm{i}\mathrm{v}\mathrm{e}\right)}$$

where ${E}_{K\left(induced\right)}$ is due to the maximum velocity induced by the merged droplet and ${E}_{K\left(effective\right)}$ is due to the velocity at which the droplet is detaching from the wall.

Energy conversion efficiency:

8 $$\upeta =\frac{{E}_{k\left(\mathrm{e}\mathrm{f}\mathrm{f}\mathrm{e}\mathrm{c}\mathrm{t}\mathrm{i}\mathrm{v}\mathrm{e}\right)}}{{E}_{K\left(\mathrm{i}\mathrm{m}\mathrm{p}\mathrm{a}\mathrm{c}\mathrm{t}\right)}}$$

**Fig. 4:**
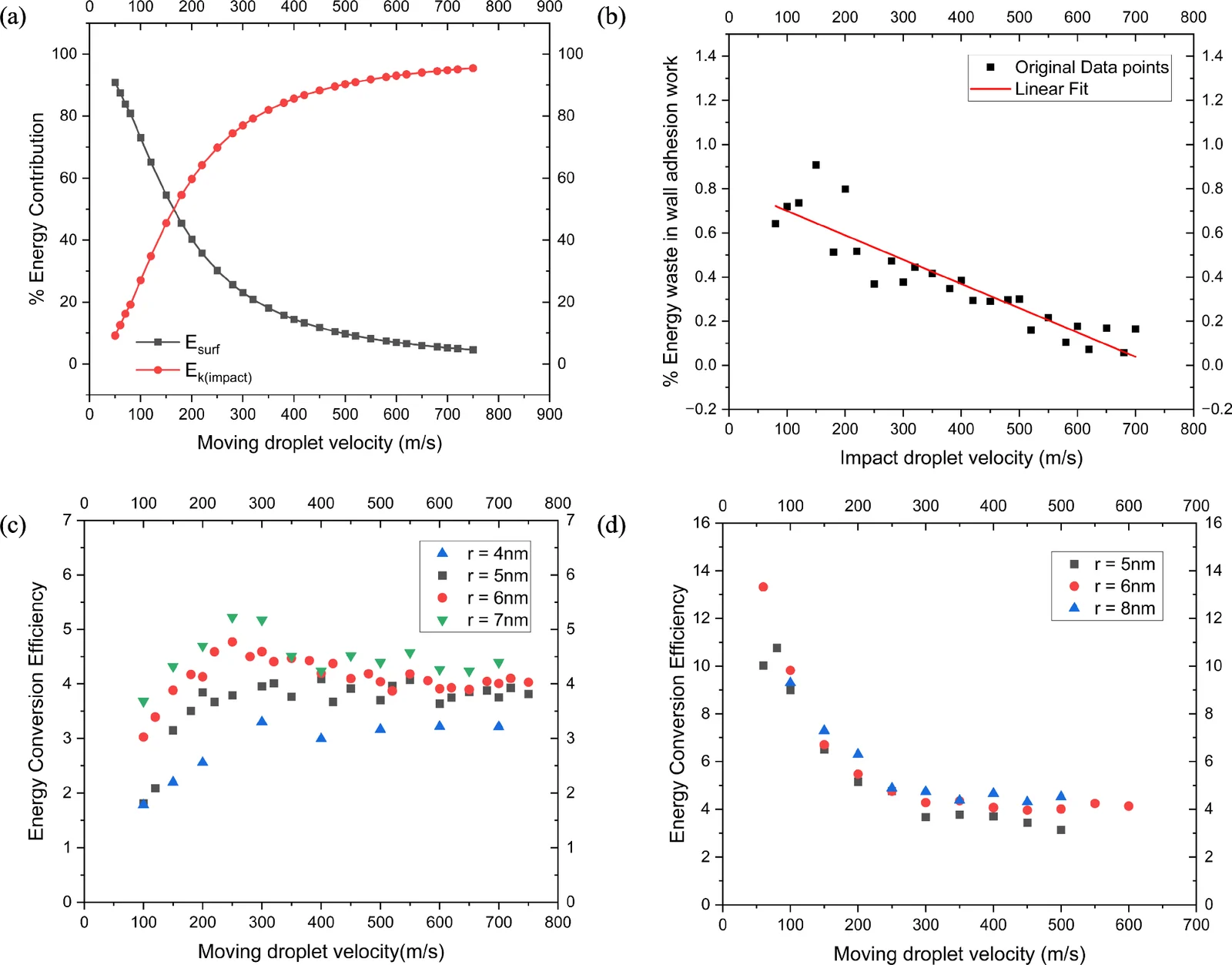
**a** For a droplet impacting another droplet, the percentage contribution of the surface energy of individual droplets and the kinetic energy of the moving droplet, on total energy, **b** percentage waste of energy to overcome surface adhesion with droplet impact velocity for 6-nm-radius droplet. **c** Percentage of total energy converted into effective kinetic energy, as a function of impact velocity for the droplet impacting another droplet, both having a radius of 4, 5, 6, and 7 nm. **d** The energy conversion efficiency for the single droplet impact having radii of 5, 6, and 8 nm

Excess surface energy coming from individual droplets ${E}_{surf}$ serves as the dominant source of energy when the impact velocity is low(<150m/s), as shown in Fig. 4 (a). The contribution of ${E}_{surf}$ is about 80 % when the impacting velocity is around 100 m/s, and it becomes negligible when the impact velocity is very high$\left(>700 m/s\right)$. At high impact velocities, the kinetic energy of the moving droplet contributes over 95% to the total energy available for conversion.

When a single droplet impacts a surface, the energy source is only the kinetic energy of the impact droplet, because after spreading, the droplet will have the exact surface area it had initially, as shown in Fig. 3a–d. No excess surface energy will come from the droplet’s surface energy as the surface area is identical while impacting and during detachment from the wall, which is shown in Fig. 3a–d, as there is no merging of droplets. So, the term ${E}_{surf}$ is zero for the scenario of a single droplet impact. However, the energy dissipation is similar to the droplet impacting another droplet case, which is surface adhesion and viscous dissipation. Also, for single droplet impact, the expression of effective kinetic energy is:

9 $${E}_{K\left(\mathrm{e}\mathrm{f}\mathrm{f}\mathrm{e}\mathrm{c}\mathrm{t}\mathrm{i}\mathrm{v}\mathrm{e}\right)}=\frac{4}{6}\uppi \uprho {r}^{3}{V}_{j}^{2}$$

Figure 4b shows how the percentage of energy dissipation by surface adhesion is influenced by the moving droplet velocity for a droplet impacting another droplet. The percentage of energy lost to overcome surface adhesion linearly decreases with the increase in impacting velocity. At high impact velocity, the droplet has so much kinetic energy that it immediately detaches from the surface, disregarding the surface adhesion. Although the contribution to energy dissipation by surface adhesion is very small, approximately 1 % at lower impact velocities, it is the crucial factor that terminates the droplet jumping when the impact droplet hits with lower kinetic energy.

Figure 4c illustrates the energy conversion efficiency with the impact velocity. It indicates the portion of surface energy after spreading, transformed into kinetic energy during the reshaping process. Energy conversion efficiency is low at lower impact velocity. It is because of the higher adhesive work at a lower impact velocity.

Maximum efficiency is noticed between 200 and 300 m/s impact velocity for all droplet sizes shown in Fig. 4c. In this range, the droplet comes to a proper round shape after spreading and immediately before leaving the surface, as shown in Fig. 3g. That is why all the surface energy is properly released just before the jumping is about to happen and converted into kinetic energy. Also, adhesive work is quite low in this range. At high impact velocity, the merged droplet leaves the surface with an unstable shape, i.e., before achieving a perfectly spherical shape as shown in Fig. 3k, and not all the surface energy is utilized during the detachment of the merged droplet. But this efficiency change is very small, and for the whole process, efficiency can be approximated as the constant value of efficiency achieved at high impact velocities. For instance, energy conversion efficiency for 6-nm-radius droplet impact is almost constant at about 4 %. A small upward shift of the energy conversion efficiency curve is noticed in Fig. 4c with the increase in droplet size because, for smaller droplets, energy waste in viscous dissipation is more as the Oh number increases with the droplet size decrease (due to the increase of viscous effect over inertia and capillary effect).

**Fig. 5:**
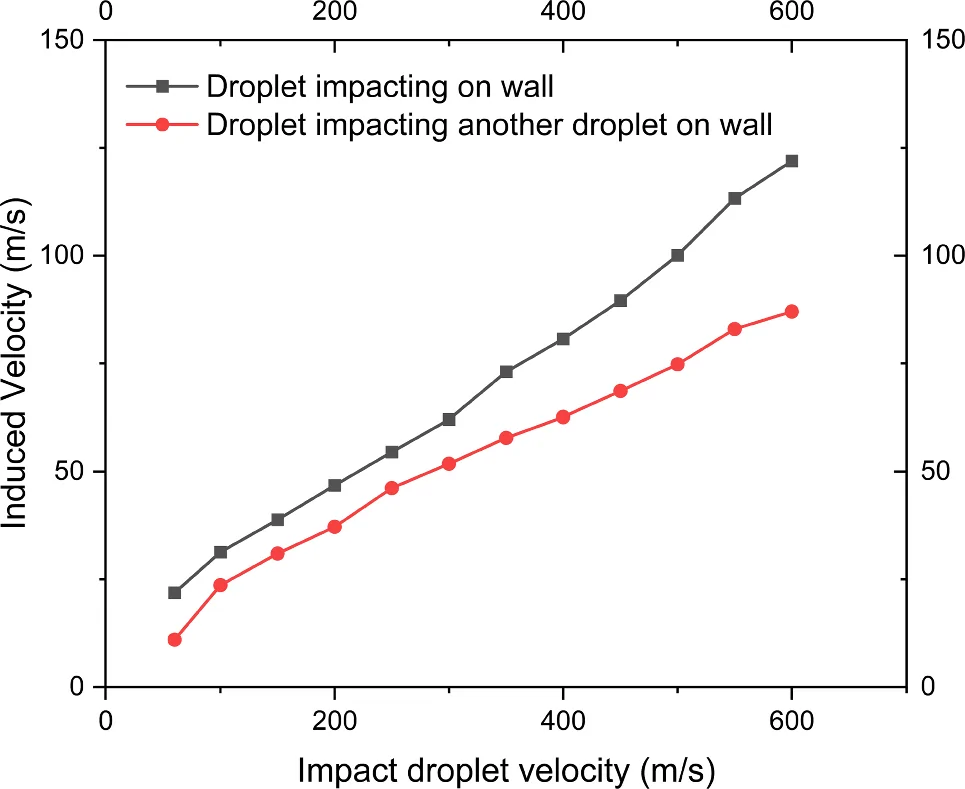
Comparison of the induced velocity as a function of impact velocity for a single droplet impacting directly on the wall and for a droplet impacting on another droplet on the wall. In both cases, the droplet radius is 6 nm

Figure 4d illustrates the energy conversion efficiency for the case of a single droplet impact. The conversion efficiency is higher than that of the double droplet case at lower impact velocity. But, at higher impact velocity, the conversion efficiency for both cases of the droplet impact becomes very close. For a droplet impacting another similar-sized droplet, the droplet volume becomes double after impact, and the spreading diameter is greater than that of a single droplet, thus, the merged droplet has to work more against the adhesion of the wall while reshaping from the spread form than the case of a single droplet impact, for the same impact velocity. Also, direct impact on a stiff surface gives a high reaction force to the response of the impact. On the other hand, when the moving droplet impacts another stationary droplet first, and then the merged droplet hits the surface with less impact force, less velocity induction results, which is shown in Fig. 5. That is why the energy conversion efficiency of a single droplet impact is about 10% whereas it is around 5% for a double droplet collision when the impact velocity is moderate. But, at high impact velocities, the energy conversion efficiency of single droplet impact falls to 4%, as shown in Fig. 4d. As discussed earlier for the case of double droplet collision, at high impact velocities, droplets do not assume a stable shape before detachment. In the case of a single droplet impact, the surface area of this unstable shape will be greater than that of its initial stage. Consequently, some of the energy becomes unavailable for conversion. Since the only source of energy is the kinetic energy of the moving droplet, the unavailability of energy due to the unstable shape significantly affects this process. This is the reason for the drastic drop in the energy conversion efficiency in Fig. 4d. At low impact velocity, the single droplet has enough time to attain a stable spherical shape before detaching from the surface.

### Effects of impact velocity, droplet size, surface roughness, and wettability

**Fig. 6:**
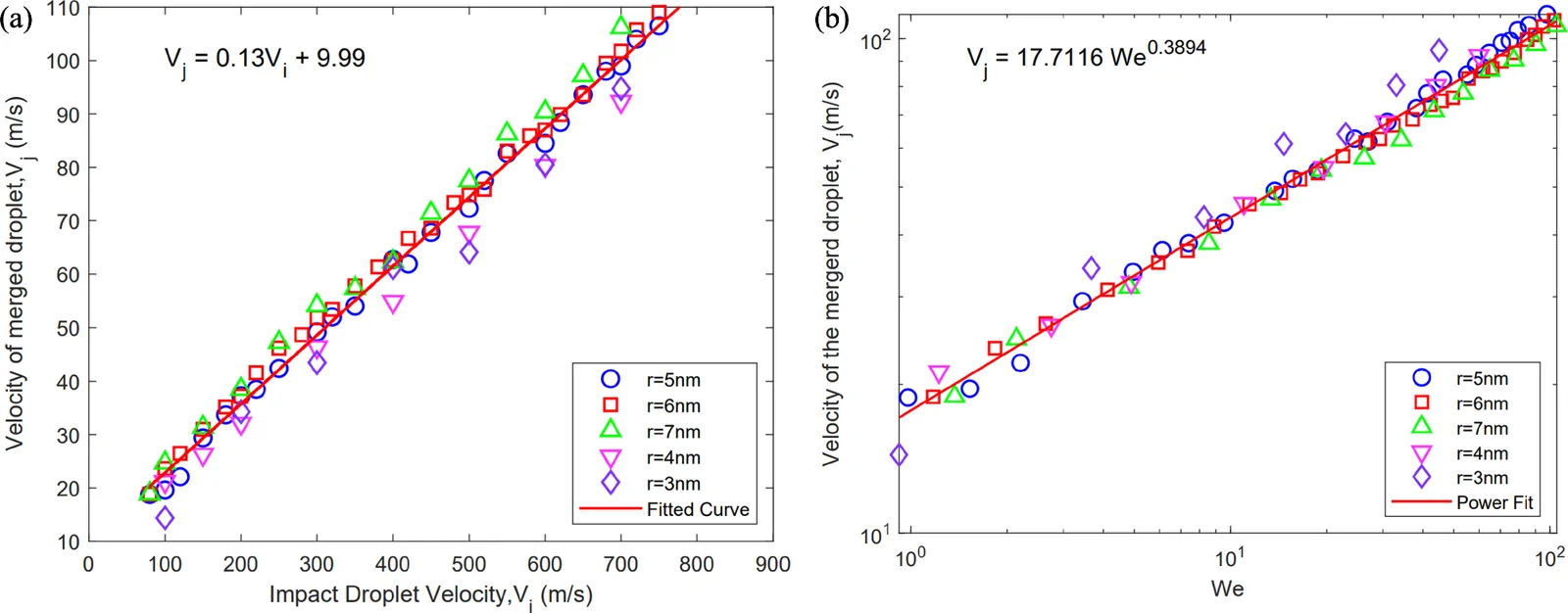
Induced velocity of the merged droplet as a function of **a** impacting velocity and **b** We number

The induced velocity $\left({V}_{ind}\right)$ is a strong function of the moving droplet velocity. The induced velocity is the velocity of the merged droplet after the collision. Figure 6a illustrates that the induced velocity of the merged droplet varies linearly with the moving droplet velocity. Induced velocities of droplets having different sizes increase with the same slope of 0.13 with impact velocity. This phenomenon justifies that the conversion efficiency is nearly constant at a certain value, as shown in Fig. 4c at moderate to high impact velocities. Because, at impact velocities over 200 m/s, the kinetic energy of the moving droplet dominates and almost single-handedly controls the whole process. The velocity of the moving droplet is expressed in terms of a dimensionless number called We number in Fig. 6b and how induced velocity increases with We number is shown. Here the We number is determined only considering the moving droplet because the droplet situated on the wall has no velocity initially. The curve of Fig. 6b is plotted in a log–log graph and all five data sets are fitted by the power-fitted curve, where the slope of the curve is 0.3894. So, the induced velocity of the merged droplet follows the following relation with the We number:

10 $${V}_{ind}\sim W{e}^{0.3894}$$

Additionally, Fig. 6a illustrates a larger induced velocity for larger droplet sizes at any impact velocity. Because the energy conversion efficiency is relatively low for smaller droplets due to greater viscous dissipation, as discussed in the previous section.

The cessation of induced velocity occurs at low impact velocities for two reasons. One obvious reason is the low kinetic energy of the impact droplet, which is insufficient to overcome viscous dissipation and adhesion forces. Another reason is the detachment of the stationary droplet from the wall just before the collision, which is caused by the attraction of the moving droplet approaching the stationary one. This phenomenon is observed for droplets making contact angles of approximately 180 degrees with the surface, indicating a pure superhydrophobic surface. In such cases, the merging occurs at a distance above the wall, and the merged droplet strikes the wall with shallow velocity, preventing it from jumping after impact. Consequently, the deformation or spreading of the merged droplet is minimal. However, at higher impact velocities, the stationary droplet does not have sufficient time to leave the surface before the collision. Nonetheless, the attraction of the moving droplet toward the stationary one is always there.

**Fig. 7:**
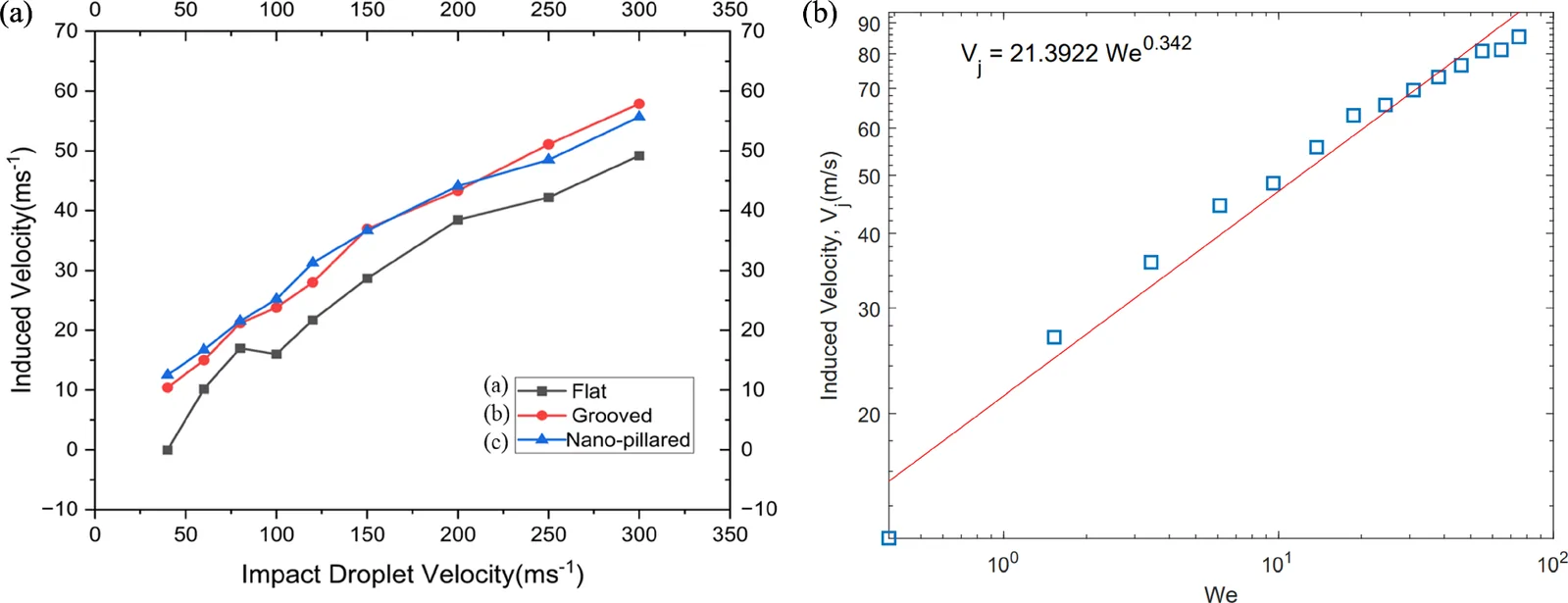
**a** Variation in induced velocity on differently structured surfaces: flat, grooved, and nano-pillared surfaces. **b** Induced velocity as a function of We number for droplet radius 5 nm and the stationary droplet is on a surface containing nano-pillars

The surface roughness also influences the jumping dynamics, similar to the single droplet impact, as highlighted in a previous study [36]. Fig. 7a depicts how velocity curves shift upward with the increase in surface roughness by creating grooves and nano-pillars on the surface. Vahabi et al. [57] showed the conversion efficiency from excess surface energy to kinetic energy can be increased by around 500% by creating a ridge on the surface in case of coalescence-induced jumping. This occurs due to the redirection of the velocity field inside the droplet. It is also applicable in droplet impact, and this phenomenon justifies the increase in velocity observed in the case of droplet impact after creating some nano-structures on the surface. Figure 7b illustrates how induced velocity increases with the increase in the We number on a surface containing nano-pillars when the droplet radius is 5 nm. The fitted curve gives a slope of 0.3420, whereas for the flat surface, it is 0.3894. So, the slope for the impact on a droplet situated on a flat surface is steeper than that of a rough surface. It is because, at lower impact velocity, the flat surface greatly affects the jumping process due to surface adhesion, the influence of which declines with impact velocity increase. However, in the case of rough surfaces, the adhesion effect is already low even for lower impact velocity. This is because, on the rough surfaces, the droplets have points where it does not make contact with the surface. Therefore, for a droplet impacting a rough surface, the effect of surface adhesion increase or decrease does not affect the induced velocity much. This is another reason why the induced velocity curve shifts upward when the surface has roughness, as shown in Fig. 7a. That is why the slope for the flat surface is steeper, but the difference in slope is small, and the scaling law (10) can also be applied to the droplet impact on the rough surface.

**Fig. 8:**
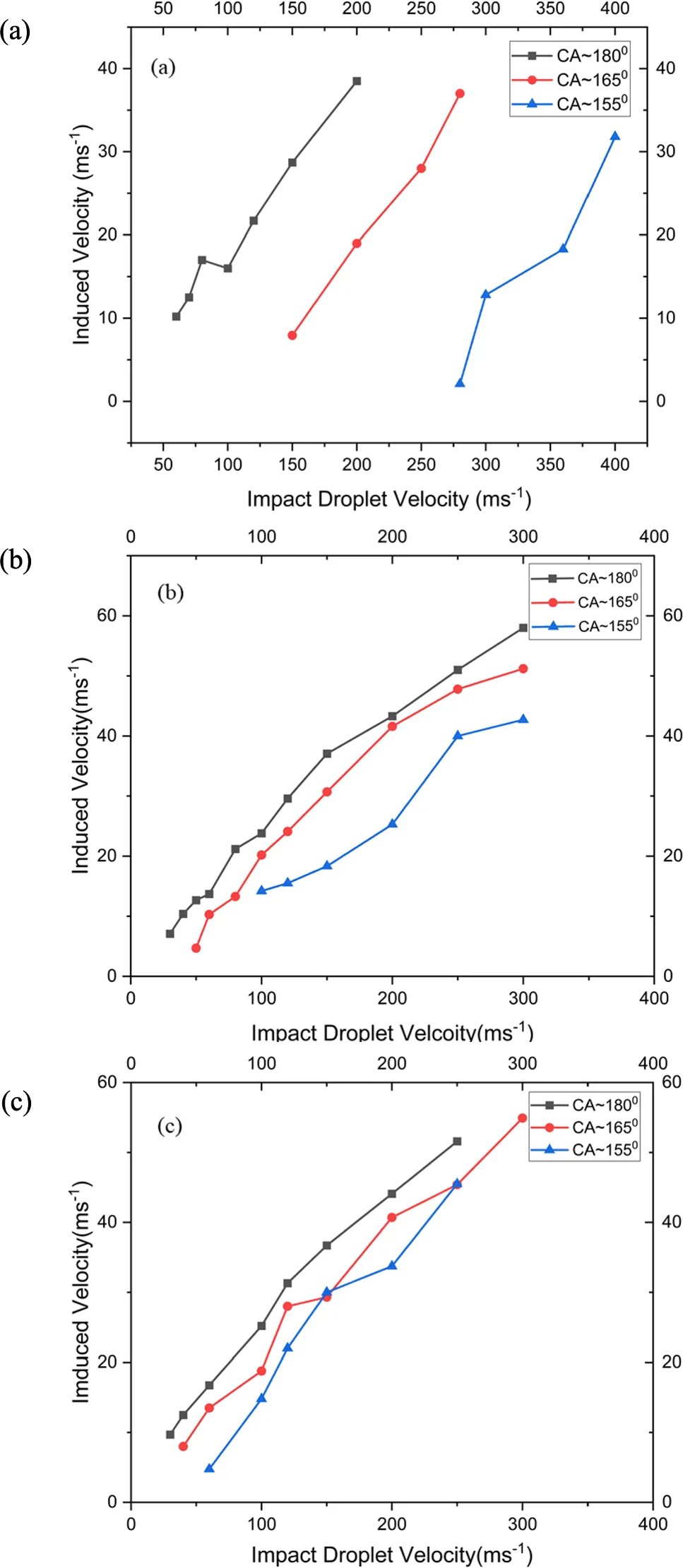
Variation in induced velocity on different structured surfaces: **a** flat, **b** grooved, and **c** nano-pillared having different wettability

The jumping velocity of the merged droplet is also influenced by surface wettability. It is easier to detach the droplet from a surface with higher hydrophobicity. Figure 8 shows how the induced velocity for a 5-nm-radius droplet responds as surface wettability changes, for smooth and rough surfaces. In the case of a flat surface, induced velocity is strongly affected by the change in contact angle (CA) between the wall and the droplet as shown in Fig. 8a. When the CA is close to 180 degrees, indicating a pure superhydrophobic surface, jumping occurs when the impact velocity is above 50 m/s. However, when the CA is reduced to near ∼ 165 and ∼ 155 degrees, to make the droplet jump from the surface, the impact droplet must have a velocity over 150 and 280 m/s, respectively. In contrast, the velocity curves of Fig. 8b and c are very close to each other for different contact angles (CAs). CAs shown in Fig. 8b and c indicate the CA a droplet would make if the surface were flat. As mentioned previously, surface roughness causes the droplet to make contact with fewer points on the surface, thereby increasing the hydrophobicity of the surface. That is why a flat surface where the droplet is making, for example, a contact angle of approximately 155 degrees, will exhibit a higher contact angle if nano-structures are constructed on the same surface.

### Modified scaling laws

#### Maximum spreading time

**Fig. 9:**
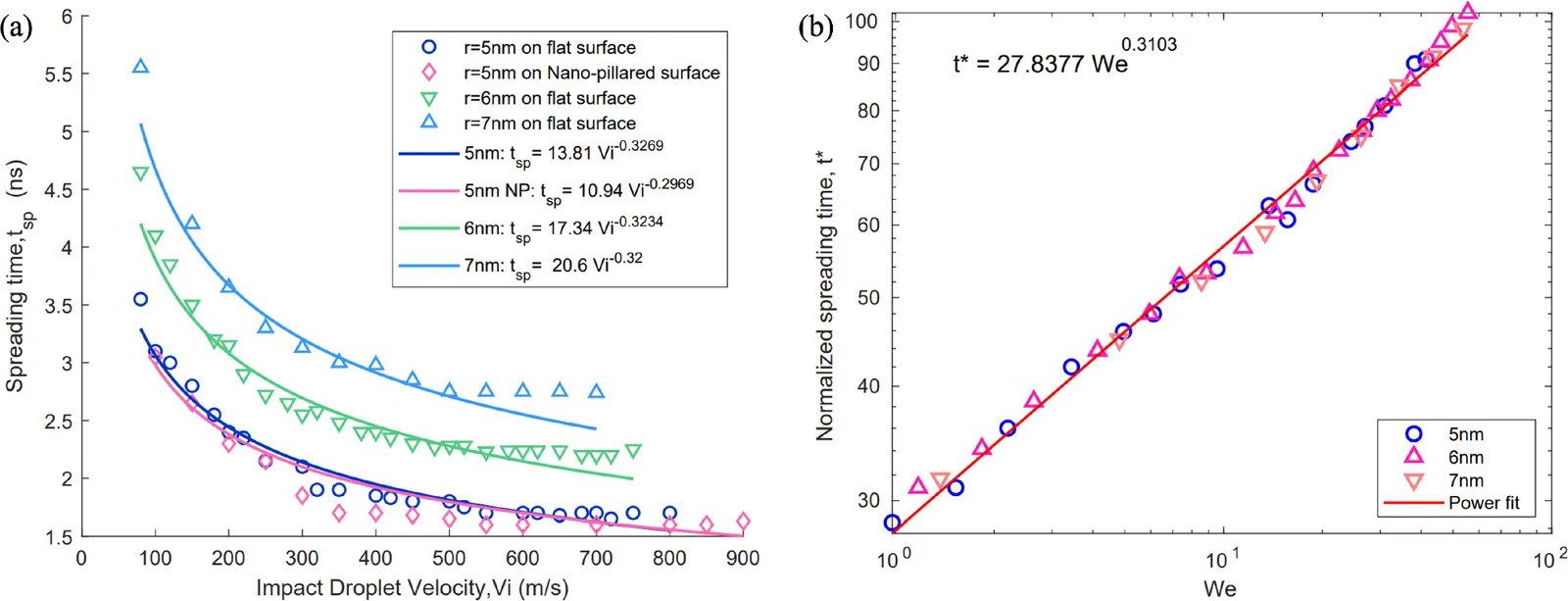
**a** Variation of spreading time as a function of impacting velocity for droplets having radius 5, 6, and 7 nm on the flat surface and 5-nm-radius droplet on a surface containing nano-pillars (NP). **b** Variation of normalized spreading time as a function of We number. Data points are fitted for 5-, 6-, and 7-nm-radius droplets on a flat superhydrophobic surface (CA ∼ 180)

After impact, the merged droplet expands across the surface until it reaches a maximum diameter. For single droplet impact, this spreading time is independent of impact velocity and it can be expressed as the following scaling law [37]: ${t}_{sp}\sim \left({D}_{o}/{V}_{o}\right)W{e}^{2/5}$. Gao et al. [36] showed that spreading time reaches a constant value at a high impact velocity, and they also found a similar relation as above. In this study of a stationary droplet on a surface being impacted by another droplet, these relations will change. For a single droplet impact, the spreading time denotes the duration of the droplet remaining in contact with the surface from the moment of impact until it detaches. But in this context, one droplet is already on the surface, and the spreading time is considered as the interval starting from when the moving droplet comes into contact with the stationary droplet until the entire merged droplet detaches from the surface. Figure 9a illustrates how spreading time varies with the velocity of the moving droplet. Comparable to the scenario of a single droplet impact, spreading time is no longer dependent on impact velocity at high velocities in this scenario as well. According to Fig. 9a, the velocity at which the spreading time becomes independent of the velocity of the impacting droplet is approximately 400 m/s. Additionally, all four cases (5-nm-, 6-nm-, and 7-nm-radius droplets on a flat surface, and the 5-nm-radius droplets on a nano-pillared surface) depicted in Fig. 9a exhibit the same trend. Equations of the power-fitted curve for individual data sets are also provided in Fig. 9a. So, the droplets follow the following relationship between spreading time and impact velocity, for the present case of droplet impact:

11 $${t}_{sp}\approx 3r{V}_{i}^{-0.32}$$

where r is the radius of the droplet and ${V}_{i}$ is the impacting velocity of the moving droplet. In the case of droplet impact on a rough surface, the spreading time does not exactly follow the Eq. (11), which is depicted in Fig. 9a for the 5-nm-radius droplet on nano-pillars, but the behavior is quite similar.

Figure 9b illustrates how normalized spreading time varies with the We number. The region of the We number where the spreading time is constant is disregarded in Fig. 9b. Maximum spreading time, ${t}^{*}$ is normalized in the following way:

12 $${t}^{*}=\frac{{V}_{i}}{D}{t}_{sp}$$

where D is the diameter of the droplets before impact and ${V}_{i}$ is the velocity of the moving droplet. ${t}^{*}$ varies similarly with the We number for the droplets of all sizes shown in Fig. 9b. So, the modified scaling law is:

13 $${t}^{*}\sim W{e}^{0.31}$$

#### Spreading factor

Another important parameter that characterizes the dynamics of impact droplet is the spreading factor which is the ratio of the initial diameter of the moving droplet to the maximum diameter achieved by the droplet after spreading on the surface ${\upbeta}_{max}={D}_{max}/{D}_{o}$.

The correlation for single droplet impact given by Wang et al. [37] for how ${\upbeta}_{\mathrm{m}\mathrm{a}\mathrm{x}}$ varies with We and Re number (${\upbeta}_{\mathrm{m}\mathrm{a}\mathrm{x}}\sim W{e}^{1/5}$ and ${\upbeta}_{\mathrm{m}\mathrm{a}\mathrm{x}}\sim W{e}^{2/3}.R{e}^{-1/3}$) will change in the present case of droplet impact onto another stationary droplet. For this study, the initial droplet diameter is taken as the diameter of the single droplet, and the maximum spreading diameter is the diameter of the merged droplet after spreading.

**Fig. 10:**
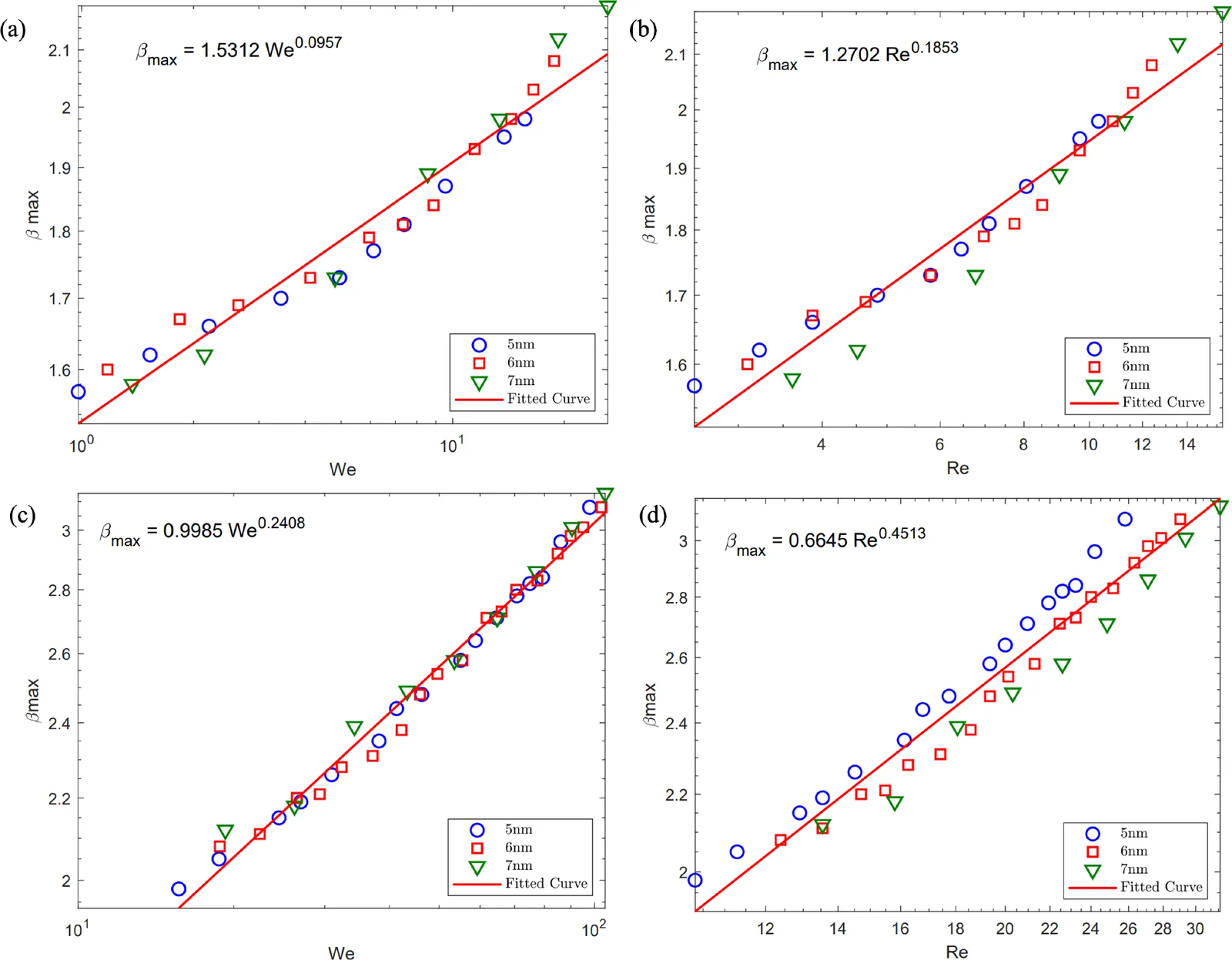
Spreading factor, βmax as a function of We and Re number in **a**, **b** low We and Re number regime and **c**, **d** high We and Re number regime. The data points that are fitted for the droplet radius 5, 6, and 7 nm on a flat superhydrophobic surface (CA ∼ 180)

As presented in the previous subsection, the spreading time is not influenced by the impact velocity for a range of We numbers and is dependent on impact velocity for another range. Also, Fig. 4c shows that energy conversion efficiency becomes constant after an impact velocity range. Considering these factors, the behavior of the spreading factor is observed in two regimes of We number: high and low We number regimes. Separate relations of the spreading factor with We and Re are found depending on We number regimes. The power-fitted curves of Fig. 10 illustrate how the spreading factor changes with the We number and Re number. At low We, the ${\upbeta}_{\mathrm{m}\mathrm{a}\mathrm{x}}$ vary with We and Re linearly in the log–log graph, making a slope of 0.1 and 0.19 approximately, as depicted in Fig. 10a and b. So, ${\upbeta}_{\mathrm{m}\mathrm{a}\mathrm{x}}$ follows scaling law ${\upbeta}_{\mathrm{m}\mathrm{a}\mathrm{x}}\sim W{e}^{0.1}$ at a low We number regime if only the We number is considered. Similarly, at a high We number regime, ${\upbeta}_{max}$ varies with We and Re, making slopes 0.24 and 0.45, respectively, as shown in Fig. 10c and d. The power of the Re number is found to be nearly double the power of the We number in both high and low We number regimes.

**Fig. 11:**
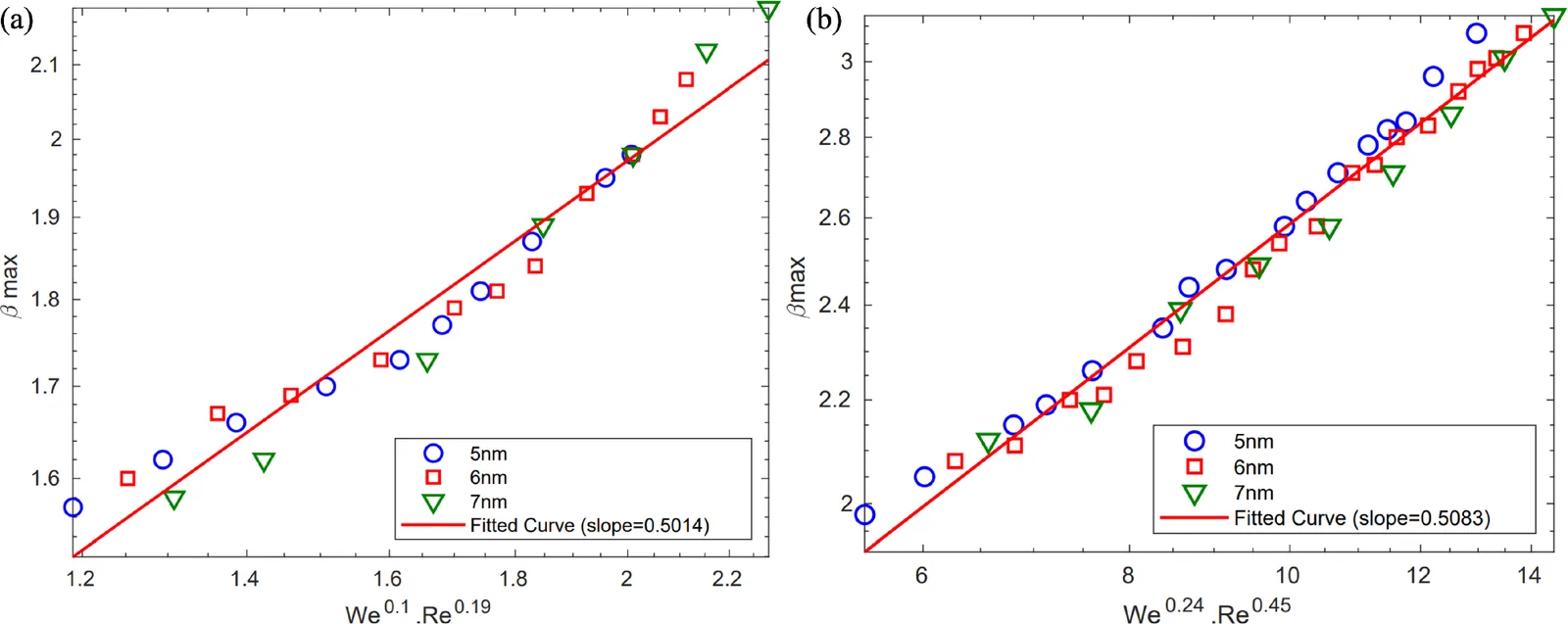
Variation of spreading factor, *β*_*max*_ with both We and Re number **a** for low We number regime and **b** for the high We number regime. Three data sets shown in the graph are for droplets having radii of 5, 6, and 7 nm on a flat superhydrophobic surface (CA ∼ 180)

If the spreading factor is plotted against both We and Re numbers together using the slopes of We and Re numbers obtained from Fig. 10, meaning if ${\upbeta}_{\mathrm{m}\mathrm{a}\mathrm{x}}$ is plotted in a log–log graph against $W{e}^{0.1}R{e}^{0.19}$ and $W{e}^{0.24}R{e}^{0.45}$ for low and high We number regimes, respectively, then the fitted curves give the slope of 0.5 for both high and low regimes of We number, as illustrated in Fig. 11. Therefore, if both We and Re numbers are considered, then the scaling law for the spreading factor for the low We number regime is:

14 $${\upbeta}_{\mathrm{m}\mathrm{a}\mathrm{x}}\sim {\left(W{e}^{0.1}R{e}^{0.19}\right)}^\frac{1}{2}$$

where 0.1 and 0.19 are the slopes of the ${\upbeta}_{max}$ vs We and Re number curves, respectively, presented in Fig. 10a and b.

For high We number:

15 $${\upbeta}_{\mathrm{m}\mathrm{a}\mathrm{x}}\sim {\left(W{e}^{0.24}R{e}^{0.45}\right)}^\frac{1}{2}$$

Similarly, where 0.24 and 0.45 represent the slopes of the ${\upbeta}_{max}$ vs We and Re number curves, respectively, shown in Fig. 10c and d. Hence, a general modified scaling law for the case of a droplet impacting upon a stationary droplet on a superhydrophobic surface is:

16 $${\upbeta}_{max}\sim {\left(W{e}^{\upalpha }\cdot R{e}^{2\upalpha }\right)}^\frac{1}{2}\sim W{e}^{0.5\upalpha }\cdot R{e}^{\upalpha }$$

where α represents the slope of the power-fitted curves of ${\upbeta}_{max}$ vs We number. α = 0.1 and 0.24 for low and high We number regimes, respectively, for a droplet impacting another droplet on a solid surface.

#### Restitution coefficient

**Fig. 12:**
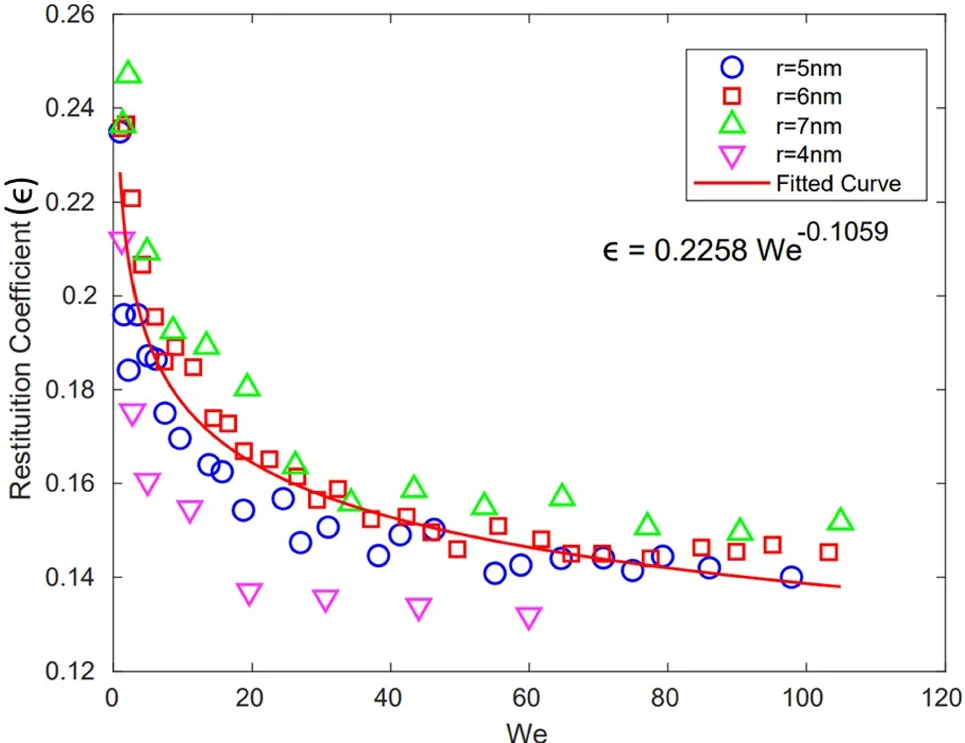
Restitution coefficient as a function of We number for droplets having radii of 4, 5, 6, and 7 nm

The restitution coefficient represents the ratio of induced velocity to impact velocity, ϵ =$\frac{{V}_{j}}{{V}_{i}}$, where ${V}_{j}$ and ${V}_{i}$ are the jumping velocity of the merged droplet and the impacting velocity of the moving droplet. The induced velocity of the merged droplet here is less than the velocity at which a single droplet departs from the surface after the impact. The reasons are described in the previous section. A study by Gao et al. [36] gives a relation between the restitution coefficient (ϵ) and the We number for a single droplet, which is$\sim W{e}^{-0.341}$. To modify this scaling law of the restitution coefficient for the present case of a moving droplet impacting a stationary droplet, how the restitution coefficient varies with the We number is presented in Fig. 12, for droplets having radii 4, 5, 6, and 7nm. ϵ follows the same trend with the We number regardless of droplet size and becomes constant at high We numbers. Hence, the power-fitted curve gives the following scaling law:

17 $$\upepsilon \sim W{e}^{-0.106}$$

As mentioned in previous sections, in the high We number regime, the kinetic energy of the moving droplet is the most dominant source of energy, and energy loss in adhesion is negligible, the only loss of energy is in viscous dissipation. Therefore, only the kinetic energy of the droplet in motion affects the process, and energy conversion efficiency is also constant at a high We number regime. Thus, the ratio of induced to impact velocity becomes almost constant at high impact velocities, as shown in Fig. 12, meaning the merged droplet induces velocity similarly to the corresponding impacting velocity of the moving droplet. Though the trend of ϵ is similar for all droplet sizes shown in Fig. 12, the value at which ϵ becomes almost constant varies with the droplet size. For instance, for high We numbers, a moving droplet having a radius of 6 nm will induce 15% of its impact velocity into the merged droplet after impact, whereas this value will be 13% for the 4-nm droplet impact.

## Conclusion

The study of impinging droplets on a stationary droplet over a solid substrate has gained considerable attention in both industrial and academic research endeavors. Understanding the underlying physics of this phenomenon is crucial for advancing its practical applications. This work studies the collision between a moving droplet and a droplet on a solid surface that is stationary, through a molecular dynamics simulation. From the simulation results, the following conclusions can be drawn:

- The process of a moving droplet colliding perpendicularly with an immobile droplet on a solid surface, leading to induced jumping, unfolds in five stages: impacting, coalescing, spreading, receding, and jumping. Insights into this process reveal its distinctions from the impact of a single droplet on the solid.
- Energy conversion lies at the heart of this phenomenon, with the kinetic energy of the moving droplet and the surface energy of the individual droplets serving as primary energy sources. A fraction of the energy (approximately 4%) is utilized to induce the jumping velocity of the merged droplet, while the rest is dissipated through adhesion work (around 1%) and viscous dissipation (approximately 95%).
- Molecular dynamics (MD) simulations conducted on surfaces with varying roughness and wettability shed light on how these factors influence the phenomenon. Induced velocity increases with surface roughness, droplet size, and surface hydrophobicity. The induced velocity curve shifts upward by almost 10 m/s when roughness is created on the surface. A decrease in contact angle for a flat surface by only 15 degrees from 180 degrees causes the minimum impact velocity for jumping to shift from 50 m/s to 150 m/s.
- Governing parameters such as maximum spreading time, spreading factor, and restitution coefficient are derived through power fitting data points obtained from MD simulations across all scenarios. Subsequent development of modified scaling laws for this phenomenon is based on these parameters. The spreading time varies linearly with $W{e}^{0.31}$ for a droplet impacting another droplet, instead of $W{e}^{0.40}$ for a single droplet impact. Similarly, the restitution coefficient has been modified from $\epsilon \sim W{e}^{-0.341}$ to $\epsilon \sim W{e}^{-0.106}$ and for spreading factor, the scaling laws are modified from ${\beta}_{max}\sim W{e}^{0.20}$ to ${\beta}_{max}\sim W{e}^{0.05}R{e}^{0.10}$ for low We numbers and from ${\beta}_{max}\sim W{e}^{2/3}R{e}^{-1/3}$ to ${\beta}_{max}\sim W{e}^{0.12}R{e}^{0.23}$ for high We number regime, by considering both We and Re numbers in both regimes.

## Data Availability

Data will be made available on request.

## References

[1] J.W.S.B. Rayleigh, *The Theory of Sound* (Macmillan, 1896)

[2] P.R. Brazier-Smith, S.G. Jennings, J. Latham, Accelerated rates of rainfall. Nature **232**, 112–113 (1971) 10.1038/232112b0 16062867

[3] K.A. Estes, I. Mudawar, Correlation of sauter mean diameter and critical heat flux for spray cooling of small surfaces. Int. J. Heat Mass Transfer **38**, 2985–2996 (1995)

[4] Z. Sun et al., Fly-eye inspired superhydrophobic anti-fogging inorganic nanostructures. Small **10**, 3001–3006 (2014) 24753310 10.1002/smll.201400516

[5] Y.-H. Kim, B. Yoo, J.E. Anthony, S.K. Park, Controlled deposition of a high-performance small-molecule organic single-crystal transistor array by direct ink-jet printing. Adv. Mater. **24**, 497–502 (2012) 22213548 10.1002/adma.201103032

[6] G. Finotello et al., The dynamics of milk droplet–droplet collisions. Exp. Fluids **59**, 17 (2017)

[7] A.L. Yarin, Drop impact dynamics: splashing, spreading, receding, bouncing…. Annu. Rev. Fluid Mech. **38**, 159–192 (2006)

[8] M.A. Quetzeri-Santiago, A.A. Castrejón-Pita, J.R. Castrejón-Pita, The effect of surface roughness on the contact line and splashing dynamics of impacting droplets. Sci. Rep. **9**, 15030 (2019) 31636321 10.1038/s41598-019-51490-5 PMC6803702

[9] R. Rabbi, A. Kiyama, J.S. Allen, T. Truscott, Droplet lift-off from hydrophobic surfaces from impact with soft-hydrogel spheres. Commun. Phys. **5**, 331 (2022)

[10] E. H. Shopnil, M. N. Azad, J. Emon, Nanodroplet Dynamics: Coalescence and Impact. Preprint at 10.48550/arXiv.2503.13659 (2025).

[11] S. Aphinyan et al., Many-body dissipative particle dynamics simulations of nanodroplet formation in 3D nano-inkjet printing. Model. Simul. Mater. Sci. Eng. **27**, 055005 (2019)

[12] J.-J. Huang, H. Huang, J.-J. Xu, Energy-based modeling of micro- and nano-droplet jumping upon coalescence on superhydrophobic surfaces. Appl. Phys. Lett. **115**, 141602 (2019)

[13] H. Wijshoff, Drop dynamics in the inkjet printing process. Curr. Opin. Colloid Interface Sci. **36**, 20–27 (2018)

[14] T. Supakar, A. Kumar, J.O. Marston, Impact dynamics of particle-coated droplets. Phys. Rev. E **95**, 013106 (2017) 28208334 10.1103/PhysRevE.95.013106

[15] R. Wen, S. Xu, X. Ma, Y.-C. Lee, R. Yang, Three-dimensional superhydrophobic nanowire networks for enhancing condensation heat transfer. Joule **2**, 269–279 (2018)

[16] C. Hao et al., Bioinspired interfacial materials with enhanced drop mobility: from fundamentals to multifunctional applications. Small **12**, 1825–1839 (2016) 26865317 10.1002/smll.201503060

[17] Y. Zhao et al., Condensate microdrop self-propelling aluminum surfaces based on controllable fabrication of alumina rod-capped nanopores. ACS Appl. Mater. Interfaces **7**, 11079–11082 (2015) 25981353 10.1021/acsami.5b03016

[18] X. Dai, B.B. Stogin, S. Yang, T.-S. Wong, Slippery wenzel state. ACS Nano **9**, 9260–9267 (2015) 26302154 10.1021/acsnano.5b04151

[19] S. Gao, Q. Liao, W. Liu, Z. Liu, Effects of solid fraction on droplet wetting and vapor condensation: a molecular dynamic simulation study. Langmuir **33**, 12379–12388 (2017) 28980811 10.1021/acs.langmuir.7b03193

[20] J. Breitenbach, I.V. Roisman, C. Tropea, From drop impact physics to spray cooling models: a critical review. Exp. Fluids **59**, 55 (2018)

[21] S. Bengaluru Subramanyam, V. Kondrashov, J. Rühe, K.K. Varanasi, Low ice adhesion on nano-textured superhydrophobic surfaces under supersaturated conditions. ACS Appl. Mater. Interfaces **8**, 12583–12587 (2016) 27150450 10.1021/acsami.6b01133

[22] J. Lv, Y. Song, L. Jiang, J. Wang, Bio-inspired strategies for anti-icing. ACS Nano **8**, 3152–3169 (2014) 24592934 10.1021/nn406522n

[23] S. Gao, Q. Liao, W. Liu, Z. Liu, Coalescence-induced jumping of nanodroplets on textured surfaces. J. Phys. Chem. Lett. **9**, 13–18 (2018) 29235875 10.1021/acs.jpclett.7b02939

[24] C.-H. Wong, Z. Dahari, A. Abd Manaf, M.A. Miskam, Harvesting raindrop energy with piezoelectrics: a review. J. Electron. Mater. **44**, 13–21 (2015)

[25] H. Wu, N. Mendel, D. Van Den Ende, G. Zhou, F. Mugele, Energy harvesting from drops impacting onto charged surfaces. Phys. Rev. Lett. **125**, 078301 (2020) 32857530 10.1103/PhysRevLett.125.078301

[26] B. Zhang, J. Li, P. Guo, Q. Lv, Experimental studies on the effect of Reynolds and Weber numbers on the impact forces of low-speed droplets colliding with a solid surface. Exp. Fluids **58**, 125 (2017)

[27] H. Fujimoto, S. Ito, I. Takezaki, Experimental study of successive collision of two water droplets with a solid. Exp. Fluids **33**, 500–502 (2002)

[28] J. de Ruiter, R. Lagraauw, D. van den Ende, F. Mugele, Wettability-independent bouncing on flat surfaces mediated by thin air films. Nat. Phys. **11**, 48–53 (2015)

[29] J. De Ruiter, F. Mugele, D. Van Den Ende, Air cushioning in droplet impact. I. Dynamics of thin films studied by dual wavelength reflection interference microscopy. Phys. Fluids **27**, 012104 (2015)

[30] J. de Ruiter, R. Lagraauw, F. Mugele, D. van den Ende, Bouncing on thin air: how squeeze forces in the air film during non-wetting droplet bouncing lead to momentum transfer and dissipation. J. Fluid Mech. **776**, 531–567 (2015)

[31] H. Xu et al., Coalescence, spreading, and rebound of two water droplets with different temperatures on a superhydrophobic surface. ACS Omega **4**, 17615–17622 (2019) 31681868 10.1021/acsomega.9b01181 PMC6822121

[32] J. De Ruiter, J.M. Oh, D. Van Den Ende, F. Mugele, Dynamics of collapse of air films in drop impact. Phys. Rev. Lett. **108**, 074505 (2012) 22401211 10.1103/PhysRevLett.108.074505

[33] E.H. Shopnil, Md.N. Azad, J. Emon, A.K.M.M. Morshed, Jumping behavior of water nanodroplets on a superhydrophobic surface in high Ohnesorge number (Oh) regime. Comput. Fluids **280**, 106344 (2024)

[34] Y.-B. Wang, X.-D. Wang, Y.-R. Yang, M. Chen, The maximum spreading factor for polymer nanodroplets impacting a hydrophobic solid surface. J. Phys. Chem. C **123**, 12841–12850 (2019)

[35] T. Koishi, K. Yasuoka, X.C. Zeng, Molecular dynamics simulation of water nanodroplet bounce back from flat and nanopillared surface. Langmuir **33**, 10184–10192 (2017) 28876073 10.1021/acs.langmuir.7b02149

[36] S. Gao, Q. Liao, W. Liu, Z. Liu, Nanodroplets impact on rough surfaces: a simulation and theoretical study. Langmuir **34**, 5910–5917 (2018) 29708343 10.1021/acs.langmuir.8b00480

[37] Y.-F. Wang et al., Scaling laws of the maximum spreading factor for impact of nanodroplets on solid surfaces. J. Fluid Mech. **937**, A12 (2022)

[38] J. Emon, B. Dindoruk, J. He, Interfacial tension suppression of confined hydrocarbons in nanopores: insights from grand canonical Monte Carlo simulations. J. Phys. Chem. C **130**, 3102–3110 (2026)

[39] J.B. Lee, D. Derome, R. Guyer, J. Carmeliet, Modeling the maximum spreading of liquid droplets impacting wetting and nonwetting surfaces. Langmuir **32**, 1299–1308 (2016) 26743317 10.1021/acs.langmuir.5b04557

[40] J. Madejski, Solidification of droplets on a cold surface. Int. J. Heat Mass Transf. **19**, 1009–1013 (1976)

[41] X.-H. Li, X.-X. Zhang, M. Chen, Estimation of viscous dissipation in nanodroplet impact and spreading. Phys. Fluids **27**, 052007 (2015)

[42] L. Moevius, Y. Liu, Z. Wang, J.M. Yeomans, Pancake bouncing: simulations and theory and experimental verification. Langmuir **30**, 13021–13032 (2014) 25286146 10.1021/la5033916

[43] C.W. Visser et al., Dynamics of high-speed micro-drop impact: numerical simulations and experiments at frame-to-frame times below 100 ns. Soft Matter **11**, 1708–1722 (2015) 25607820 10.1039/c4sm02474e

[44] B. Zhang, Q. Lei, Z. Wang, X. Zhang, Droplets can rebound toward both directions on textured surfaces with a wettability gradient. Langmuir **32**, 346–351 (2016) 26669260 10.1021/acs.langmuir.5b04365

[45] M. Choi, G. Son, W. Shim, Numerical simulation of droplet impact and evaporation on a porous surface. Int. Commun. Heat Mass Transfer **80**, 18–29 (2017)

[46] K. Yokoi, Numerical studies of droplet splashing on a dry surface: triggering a splash with the dynamic contact angle. Soft Matter **7**, 5120 (2011)

[47] W.-Z. Yuan, L.-Z. Zhang, Lattice Boltzmann simulation of droplets impacting on superhydrophobic surfaces with randomly distributed rough structures. Langmuir **33**, 820–829 (2017) 28036183 10.1021/acs.langmuir.6b04041

[48] A.P. Thompson et al., LAMMPS—a flexible simulation tool for particle-based materials modeling at the atomic, meso, and continuum scales. Comput. Phys. Commun. **271**, 108171 (2022)

[49] P. Hirel, Atomsk: a tool for manipulating and converting atomic data files. Comput. Phys. Commun. **197**, 212–219 (2015)

[50] M.N. Joswiak, R. Do, M.F. Doherty, B. Peters, Energetic and entropic components of the Tolman length for mW and TIP4P/2005 water nanodroplets. J. Chem. Phys. **145**, 204703 (2016) 27908116 10.1063/1.4967875

[51] V. Molinero, E.B. Moore, Water modeled as an intermediate element between carbon and silicon. J. Phys. Chem. B **113**, 4008–4016 (2009) 18956896 10.1021/jp805227c

[52] S. Perumanath, M.K. Borg, J.E. Sprittles, R. Enright, Molecular physics of jumping nanodroplets. Nanoscale **12**, 20631–20637 (2020) 32776062 10.1039/d0nr03766d

[53] Y.-B. Wang et al., Universal model for the maximum spreading factor of impacting nanodroplets: from hydrophilic to hydrophobic surfaces. Langmuir **36**, 9306–9316 (2020) 32697096 10.1021/acs.langmuir.0c01879

[54] L. Qiu, S. Qian, Y. Ni, Q. Tong, Optimum substrate stiffness in coalescence-induced droplet jumping. Phys. Chem. Chem. Phys. **25**, 14368–14373 (2023) 37183923 10.1039/d3cp00835e

[55] S. Perumanath, R. Pillai, M.K. Borg, Contaminant removal from nature’s self-cleaning surfaces. Nano Lett. **23**, 4234–4241 (2023) 37154913 10.1021/acs.nanolett.3c00257 PMC10214492

[56] D. Zrnić, P. Berglez, G. Brenn, Weakly nonlinear shape oscillations of a Newtonian drop. Phys. Fluids **34**, 043103 (2022)

[57] H. Vahabi, W. Wang, J.M. Mabry, A.K. Kota, Coalescence-induced jumping of droplets on superomniphobic surfaces with macrotexture. Sci. Adv. **4**, eaau3488 (2018) 30430135 10.1126/sciadv.aau3488 PMC6226286

